# The complete mitochondrial genome of smooth spooner crab, *Etisus laevimanus* Randall, 1840 (Crustacea: Decapoda: Xanthoidea) from the East Sea, Korea

**DOI:** 10.1080/23802359.2025.2559712

**Published:** 2025-09-16

**Authors:** Sang-kyu Lee, Jinsoon Park, Hyun Soo Rho, Sang-Hwa Lee, Jong Seong Khim

**Affiliations:** ^a^ School of Earth and Environmental Sciences and Research Institute of Oceanography, Seoul National University, Seoul, Republic of Korea; ^b^ Department of Convergence Study on the Ocean Science and Technology, Korea Maritime and Ocean University, Busan, Republic of Korea; ^c^ East Sea Environmental Research Center, Korea Institute of Ocean Science & Technology, Uljin, Republic of Korea; ^d^ Invertebrate Diversity Institute, Cheongju, Republic of Korea

**Keywords:** *Etisus laevimanus*, smooth spooner crab, complete mitochondrial genome, Xanthoidea, Decapoda

## Abstract

The mitogenome of the smooth spooner crab *Etisus laevimanus* Randall, 1840, was sequenced completely for the first time. The total *E. laevimanus* mitogenome length is 15,714 bp, containing 13 protein-coding genes, two ribosomal RNA genes, 22 transfer RNA genes, and one non-coding region. The gene order is consistent with that in other xanthoid species. The base composition is 32.9% A, 20.6% C, 11.6% G, and 34.9% T, with a G–C content of 32.1%. The phylogenetic analysis using the xanthoid mitogenome showed that the genus *Etisus* may not be monophyletic, as suggested by a few previous studies.

## Introduction

Brachyurans are important components of marine ecosystems, occupying various niches as commensals, primary and secondary consumers, and food for many marine species (Lee 2015). Xanthoidea is one of the most dominant Brachyuran superfamilies, with 760 species of 189 genera belonging to four of its families reported worldwide (Davie et al. 2015). The genus *Etisus* H. Milne Edwards, 1834, of the family Xanthidae MacLeay, 1838, consists of 26 black spooner crab species (Poore and Ahyong 2023). *Etisus anaglyptus* and *E. laevimanus*, known to inhabit tropical to subtropical regions such as those in India, Singapore, and Japan, have also been reported to be present near the drainage outlet of Uljin Power Plant in Korea (Lee et al. 2012, 2014).

Traditional classifications are based on morphological features related to their ecology, and their classification forms monophyletic groups. Some researchers have implied that there are distinct, monophyletic groups within certain xanthid subfamilies (Mendoza et al. 2022). Phylogenetic studies of the superfamily Xanthoidea have been performed using multigene analyses, and some reclassification into previously used families outside of the four Xanthoidea families has occurred. Furthermore, research has indicated that *E. laevimanus* and *E. guinotae* belong to the subfamily Xanthinae rather than the subfamily Etisinae (Lai et al. 2011; Mendoza et al. 2022). However, mitogenomic records of xanthoid species remain insufficient. The mitochondrial genomes of six species in this superfamily have been documented (Karagozlu et al. 2018a, 2018b; Karagozlu, Dinh, et al. 2018; Liu and Shen 2021; Wang et al. 2021).

In this study, we analyzed the complete mitogenome of the smooth spooner crab *E. laevimanus.*

The aims of the work were to contribute to a better understanding of the evolution and classification of the genus *Etisus*, and to enhance insight into the broader classification of Xanthidae within the superfamily Xanthoidea.

## Materials and methods

**Figure 1.**
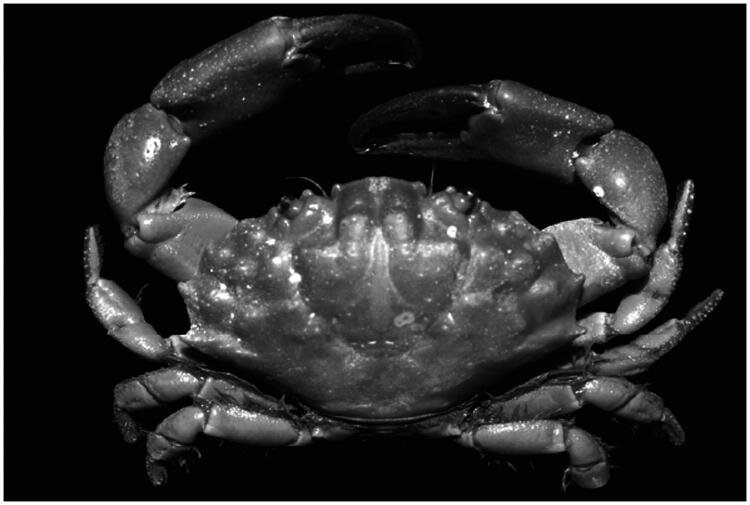
*Etisus laevimanus* Randall, 1840 (carapace width, 48.5 mm; length, 34.2 mm). Photographs were taken by S.-k. Lee.

One specimen of *E. laevimanus* (Figure 1) was collected from the subtidal zone of Uljin on the east coast of Korea on 27 September 2012 (geographic location: 37.0825 N 129.4062 E), fixed by ethyl alcohol, and deposited at the Laboratory of Marine Benthic Ecology, Seoul National University, Seoul, Republic of Korea. To ensure accurate identification, the specimen’s morphological characteristics were examined meticulously following the description of Serène (1984) and Ng (1998).

Genomic DNA was extracted from the specimens’ pereiopods, with high-purity mtDNA extraction performed using a Qproteome mitochondrial isolation kit (Qiagen, Hilden, Germany), an E.Z.N.A mollusk DNA kit (Omega Bio-Tech, Norcross, GA), a RELI-g mtDNA kit (Qiagen, Valencia, CA), and Exo-Resistant random primer (Thermo Fisher Scientific, San Francisco, CA). A DNA library was constructed using a NEXTflex^®^ rapid DNA sequencing kit (BIOO Scientific, Austin, TX). The complete mitochondrial genome was sequenced using a NovaSeq6000 platform (Illumina, San Diego, CA). After NGS reads were assembled and analyzed using two programs, Geneious v.9.1.8 (Biomatters, Auckland, New Zealand) (Kearse et al. 2012) and NOVOPlasty-4.3.3 (Dierckxsens et al. 2017). Protein-coding genes (PCGs) were annotated by identifying their open reading frames and comparing them with other reported mitogenomes of xanthoid species using the MITOS web server (Bernt et al. 2013). Ribosomal and transfer RNA genes were identified by comparison with homologous gene sequences from other xanthoid mitogenomes. DNA barcoding analysis of the genus *Etisus* was conducted using cytochrome C oxidase subunit I (COI) to accurately identify the specimens tested.

A phylogenetic analysis was performed using 17 mitogenome sequences of 16 species. The two pilumnoids were used as the outgroup. Each mitogenome sequence (13 PCG nucleotides, excluding stop codons) from a total of 16 species, including *E. laevimanus*, was aligned using MAFFT v.7 (Katoh and Standley 2013). All gene sequences were then concatenated, and a phylogenetic tree was reconstructed based on the concatenated dataset using the maximum-likelihood method with the GTR + G + I model in RaxmlGUI 2.0 (Edler et al. 2021), with bootstrap (BP) values calculated from 10,000 replicates. After phylogenetic analysis, the phylogenetic tree was modified using the TREEFINDER program (Jobb et al. 2004).

## Results

To confirm the species of *Etisus laevimanus* used in the experiment, COI sequences were obtained from the mitogenomes of 16 crab species (including the sequence of the present study) (Table S1). Partial COI sequences for three additional species (*E. anaglyptus, E. dentatus*, and *E. laevimanus*) were acquired from the NCBI (National Center for Biotechnology Information) GenBank, bringing the total to 20 COI sequences from 16 species for DNA barcoding analysis. This analysis allowed for the accurate identification of the *Etisus laevimanus* species used in the experiment (Figure S2).

**Figure 2.**
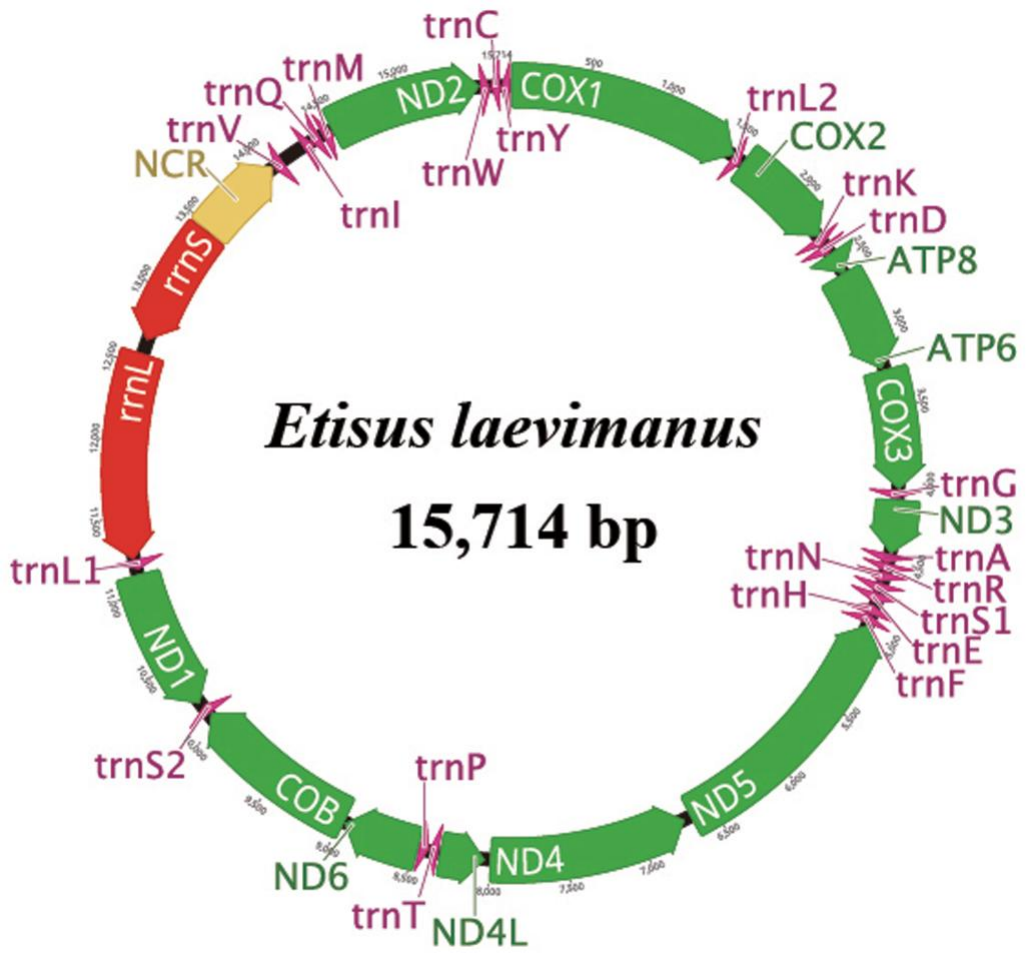
Circular representation of the complete *Etisus laevimanus* mitogenome. Arrows show the directions of transcription.

The complete circular mitogenome of *E. laevimanus* (GenBank accession no. PP239406) was 15,714 bp in length (Figure 2), consistent with published lengths for xanthoid (15–16 kb). It includes 37 genes – 13 PCGs, two ribosomal RNA genes, and 22 transfer RNA genes – and one non-coding region of 594 bp.

The mitochondrial genome’s base composition is 32.9% A, 20.6% C, 11.6% G, and 34.9% T. The lengths of the *rrnL* and *rrnS* genes in this species are 1347 and 813 bp, respectively, and those of the transfer RNAs identified range from 63 to 69 nucleotides. A putative control region (594 bp) is present between *rrnS* and *trnV*, as in other xanthoid mitochondrial genomes. Three types of PCG start codon were identified: ATA (*ATP6*, *COX3*, and *ND4*), ATG (*ATP8*, *COB*, *COX1–2*, *ND1–2*, and *ND4L*), and ATT (*ND3*, *ND5*, and *ND6*). Identified stop codons are TAA (*ATP6*, *COX3*, *ND3*, *ND5*–*6*, and *ND4L*) and TAG (*ATP8*, *ND1*–*2*, and *ND4*); three PCGs (*COX1*–*2* and *COB*) have incomplete (T) stop codons. The mitogenome coverage depth figure is detailed (Figure S1).

**Figure 3.**
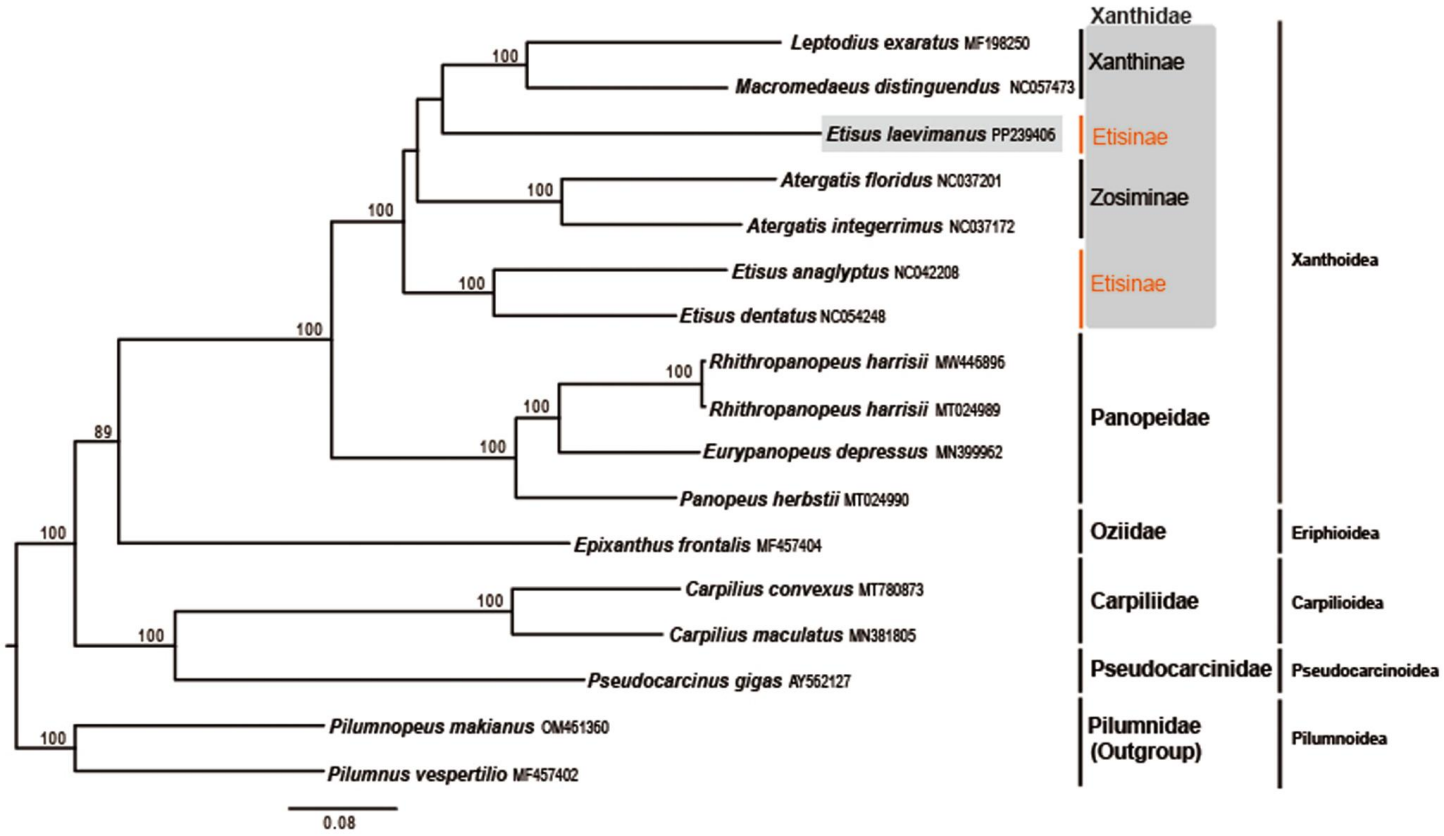
Maximum-likelihood tree based on concatenated data on 13 protein-coding gene sequence from 10 xanthoid species, one eriphioid, two carpilioids, one pseudocarcinoid, and two pilumnoids. Two pilumnoids (*Pilumnus vespertilio* and *Pilumnopeus makianus*) served as outgroups. GenBank accession numbers for the sequences are provided after corresponding species names in the tree. Bootstrap values were calculated from 10,000 replicates, and those >70% are indicated at the base of each node. Species used include the following: *Leptodius exaratus* MF198250, *Macromedaeus distinguendus* NC_057473 (Wang et al. 2021), *Etisus laevimanus* PP239406, *Atergatis floridus* NC_037201 (Karagozlu et al. 2018a), *Atergatis integerrimus* NC_037172 (Karagozlu et al. 2018b), *Etisus anaglyptus* NC_042208 (Karagozlu et al. 2018c), *Etisus dentatus* NC_054248 (Liu and Shen 2021), *Rhithropanopeus harrisii* NW446896; MT024989 (Jennings et al. 2021), *Eurypanopeus depressus* MN399962 (Jennings et al. 2021), *Panopeus herbstii* MT024990 (Jennings et al. 2021), *Epixanthus frontalis* MF457404 (Tan et al. 2018), *Carpilius convexus* MT780873, *Carpilius maculatus* MN381805 (Liu et al. 2019), *Pseudocarcinus gigas* AY562127 (Miller et al. 2005), *Pilumnopeus makianus* OM461360 (Duan et al. 2022), and *Pilumnus vespertilio* MF457402 (Tan et al. 2018).

The phylogenetic analysis suggested that *E. laevimanus* is a member of the family Xanthidae, forming a clade with *M. distinguendus* and *L. exaratus*, although this association was not strongly supported (BP value < 70; Figure 3).

## Discussion and conclusions

The phylogenetic analysis of the mitogenomes of 16 species revealed conformity to their superfamily and family (Figure 3). *Etisus laevimanus* forms a clade with *Leptodius exaratus* and *Macromedaeus distinguendus*, rather than with *E. anaglyptus* and *E. dentatus*, even though they are congeneric. As shown in the results, several previous studies suggest that the genus *Etisus* is polyphyletic (Felde and Thoma 2010; Lai et al. 2011; Lasley et al. 2013, 2015).

However, the BP value is below 70, and since only three species of the genus *Etisus* mitogenomes are available, additional data from other species are needed.

In recent studies, Mendoza et al. (2022) synonymized the subfamily *Chlorodiellinae*, which has a similar morphology to the subfamily *Etisinae*, based on the results of their molecular phylogenetic study using mitochondrial and nuclear genes. Additionally, they synonymized the genera *Leptodius*, *Macromedaeus*, and *Neoxanthops* of the subfamily *Xanthinae*. Here, *Etisus laevimanus* does not cluster with the species of the genera *Leptodius* and *Macromedaeus*, but rather groups with *E. dentatus* and *E. anaglyptus*. However, a formal morphological diagnosis for this grouping has yet to be proposed (Lasley et al. 2023).

This study contributes to the initial complete *E. laevimanus* mitogenome sequencing. Its results offer valuable insight into phylogenetic studies and the examination of this species’ phylogeography. They also provide a foundation for extensive taxonomic investigations of xanthoids, with the incorporation of this species as a representative of the genus *Etisus*.

## Supplementary Material

Supplemental Material

Supplemental Material

Supplemental Material

Supplemental Material

## Data Availability

The data that support the findings of this study are openly available in the GenBank of NCBI at https://www.ncbi.nlm.nih.gov under the accession PP239406. The associated BioProject, SRA, and Bio-sample numbers are PRJNA1071473, SRR27823246, and SAMN39685897, respectively.
