# Supplementary material for: The complete mitochondrial genome of smooth spooner crab, Etisus laevimanus Randall, 1840 (Crustacea: Decapoda: Xanthoidea) from the East Sea, Korea
Source: Mitochondrial DNA B Resour. 2025 Sep 16;10(10):963–6. doi: 10.1080/23802359.2025.2559712 (PMC12444958; doi:10.1080/23802359.2025.2559712)
Supplement: Supplemental Material [file TMDN_A_2559712_SM6337.docx]

<Supplementary data>

The complete mitochondrial genome of smooth spooner crab, *Etisus laevimanus* Randall, 1840 (Crustacea: Decapoda: Xanthoidea) from the East Sea, Korea

Sang-kyu Lee^a^, Jinsoon Park^b^, Hyun Soo Rho^c^, Sang-Hwa Lee^d^ and Jong Seong Khim^a,*^

***Table of Contents***

**Supplementary figures**

**Figure S1.** Read coverage depth map of *Etisus laevimanus* (PP239406) created in Geneious Prime v.9.1.8 (<https://www.geneious.com>). ----------------------------------------------------------------------------------------- S2

**Figure S2.** Maximum likelihood (ML) tree inferred from COI sequences for DNA barcoding analysis. Catalogue numbers are included. Numbers indicate maximum likelihood bootstrap support; values below 70 are not shown. ------------------------------------------------------------------------------------------------------------------- S3

**Supplementary tables**

**Table S1.** The list of crab species used to construct the maximum-likelihood tree from the 13 protein-coding genes and the COI gene sequences.----------------------------------------------------------------------------------------S4

* Corresponding author.

Address: School of Earth and Environmental Sciences & Research Institute of Oceanography,

Seoul National University, 1 Gwanak-ro, Gwanak-gu, Seoul 08826, Republic of Korea.

Tel.: +82 2 880 6750.

E-mail addresses: [jskocean@snu.ac.kr](mailto:jskocean@snu.ac.kr) (J.S. Khim).


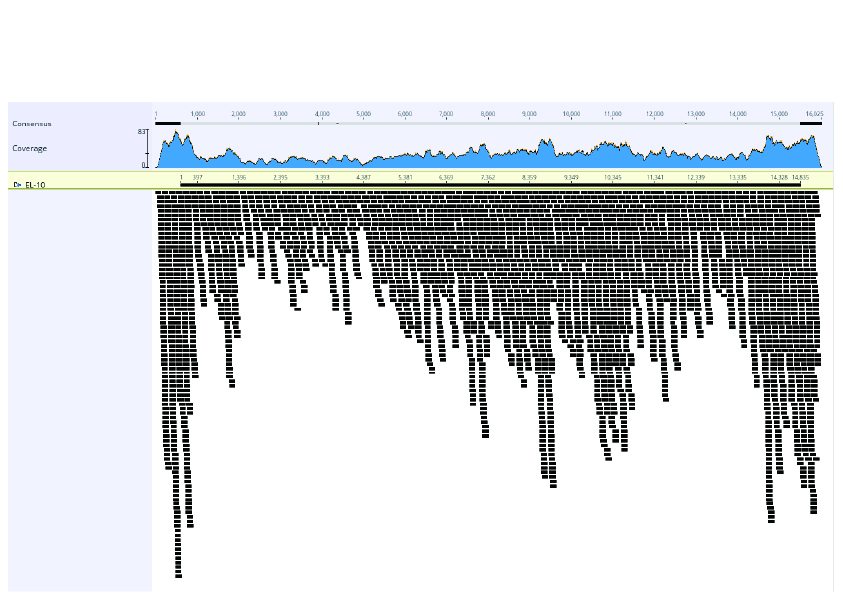


Figure S1. Read coverage depth map of *Etisus laevimanus* (PP239406) created in Geneious Prime v.9.1.8 (https://www.geneious.com)


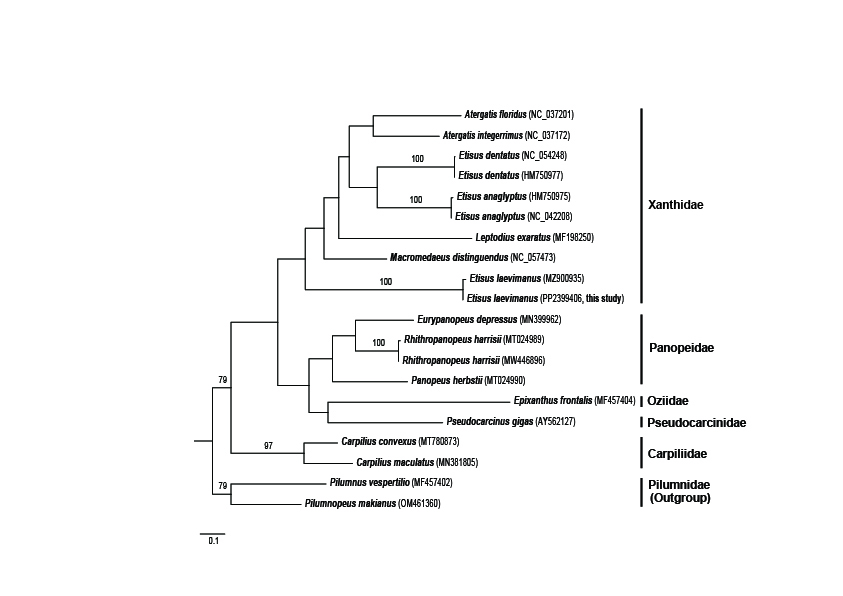


Figure S2. Maximum likelihood (ML) tree inferred from COI sequences for DNA barcoding analysis. Catalogue numbers are included. Numbers indicate maximum likelihood bootstrap support; values below 70 are not shown. Addtinoal Species data used include the following: *Etisus anaglyptus* HM750975 (Mendoza et al., 2022), *Etisus dentatus* HM750977 (Mendoza et al., 2022), *Etisus laevimanus* Yuan et al., 2022.

Table S1. The list of crab species used to construct the maximum-likelihood tree on the 13 protein-coding genes and the COI gene sequences.

| Taxon name | GenBank No. | Reference |
| --- | --- | --- |
| Family Xanthidae |  |  |
| *Atergatis floridus* (Linnaeus, 1767) | NC_037201 | Karagozlu et al. 2018a |
| *Atergatis integerrimus* (Lamarck, 1818) | NC_037172 | Karagozlu et al. 2018b |
| *Etisus anaglyptus* H. Milne Edwards, 1834 | NC_042208 | Karagozlu et al. 2018c |
| *Etisus anaglyptus* H. Milne Edwards, 1834 | HM750975 | Mendoza et al., 2022 |
| *Etisus dentatus* (Herbst, 1785) | NC_054248 | Liu & Shen 2021 |
| *Etisus dentatus* (Herbst, 1785) | HM750977 | Mendoza et al., 2022 |
| *Etisus laevimanus* Randall, 1840 | PP239406 | Karagozlu et al. 2018a |
| *Etisus laevimanus* Randall, 1840 | MZ900935 | Yuan et al., 2022 |
| *Leptodius exaratus* (H. Milne Edwards, 1834) | MF198250 | Wang et al. 2021 |
| *Macromedaeus distinguendus* (De Haan, 1835) | NC_057473 | Wang et al. 2021 |
| Family Panopeidae Ortmann, 1893 |  |  |
| *Eurypanopeus depressus* (Smith, 1869) | MN399962 | Jennings et al. 2021 |
| *Panopeus herbstii* H. Milne Edwards, 1834 | MT024990 | Jennings et al. 2021 |
| *Rhithropanopeus harrisii* (Gould, 1841) | NW446896 | Jennings et al. 2021 |
| *Rhithropanopeus harrisii* (Gould, 1841) | MT024989 | Jennings et al. 2021 |
| Family Oziidae Dana, 1851 |  |  |
| *Epixanthus frontalis* (H. Milne Edwards, 1834) | MF457404 | Tan et al. 2018 |
| Family Carpiliidae Ortmann, 1893 |  |  |
| *Carpilius convexus* (Forskål, 1775) | MT780873 | Liu, Yang, He 2019 |
| *Carpilius maculatus* (Linnaeus, 1758) | MN381805 | Liu, Yang, He 2019 |
| Family Pseudocarcinidae Ng & Davie, 2020 |  |  |
| *Pseudocarcinus gigas* (Lamarck, 1818) | AY562127 | Miller et al. 2005 |
| Family Pilumnidae Samouelle, 1819 |  |  |
| *Pilumnopeus makianus* (Rathbun, 1931) | OM461360 | Duan et al. 2022 |
| *Pilumnus vespertilio* (Fabricius, 1793) | MF457402 | Tan et al. 2018 |

Additinoal references

Yuan Z, Jiang W, Sha Z. 2022. A review of the common crab genus *Macromedaeus* Ward, 1942 (Brachyura, Xanthidae) from China Seas with description of a new species using integrative taxonomy methods. PeerJ 10:e12735. DOI: 10.7717/peerj.12735.

Mendoza JC, Chan KO, Lai JC, Thoma BP, Clark PF, Guinot D, Felder DL., Ng PKL. 2022. A comprehensive molecular phylogeny of the brachyuran crab superfamily Xanthoidea provides novel insights into its systematics and evolutionary history. Molecular Phylogenetics and Evolution, 177, 107627. DOI: 10.1016/j.ympev.2022.107627.

Karagozlu MZ, Dinh TD, Nguyen VQ, Kim CB. 2018. Analysis of complete mitochondrial genome of *Etisus anaglyptus* (Arthropoda, Decapoda, Xanthidae) with phylogenetic consideration. Mitochondrial DNA Part B, 3: 1, 278-279. DOI: 10.1080/23802359.2018.1443038.
