# Supplementary figures and images for: The complete mitochondrial genome of smooth spooner crab, Etisus laevimanus Randall, 1840 (Crustacea: Decapoda: Xanthoidea) from the East Sea, Korea
Source: Mitochondrial DNA B Resour. 2025 Sep 16;10(10):963–6. doi: 10.1080/23802359.2025.2559712 (PMC12444958; doi:10.1080/23802359.2025.2559712)

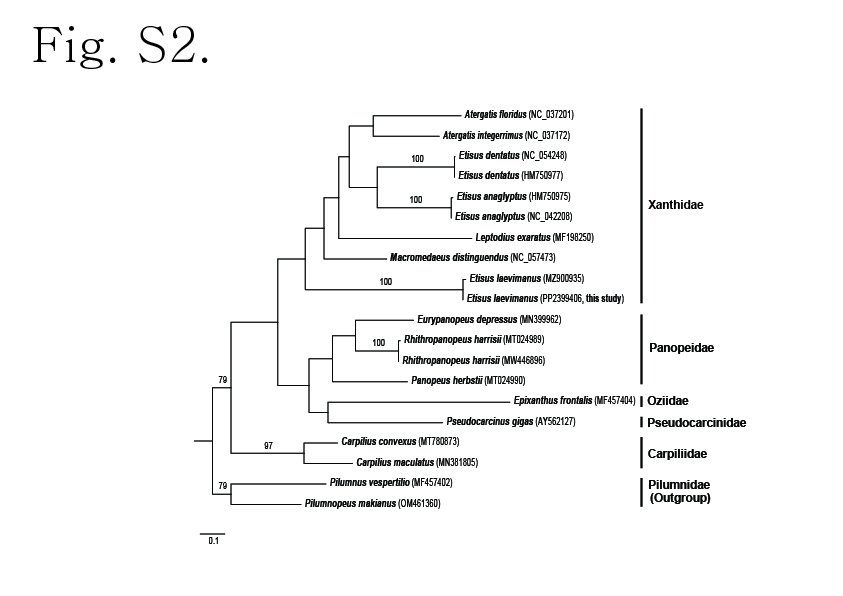

Supplement: Supplemental Material [file TMDN_A_2559712_SM6331.jpg]

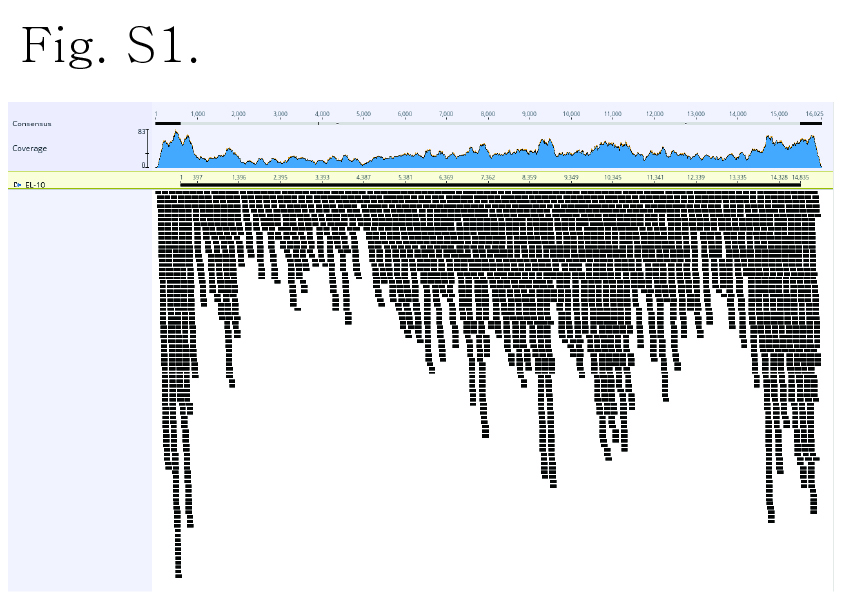

Supplement: Supplemental Material [file TMDN_A_2559712_SM6327.jpg]
